# Design of nonstandard computational method for stochastic susceptible–infected–treated–recovered dynamics of coronavirus model

**DOI:** 10.1186/s13662-020-02960-y

**Published:** 2020-09-18

**Authors:** Wasfi Shatanawi, Ali Raza, Muhammad Shoaib Arif, Kamaledin Abodayeh, Muhammad Rafiq, Mairaj Bibi

**Affiliations:** 1 Department of Mathematics and General Sciences, Prince Sultan University, Riyadh, Saudi Arabia; 2 Department of Mathematics, Stochastic Analysis & Optimization Research Group, Air University, PAF Complex E-9, Islamabad, 44000 Pakistan; 3 Department of Mathematics, National College of Business Administration and Economics, Lahore, Pakistan; 4 Department of Medical Research, China Medical University Hospital, China Medical University, Taichung, 40402 Taiwan; 5 Department of Mathematics, Hashemite University, Zarqa, Jordan; 6 Faculty of Engineering, University of Central Punjab, Lahore, 54500 Pakistan; 7 Department of Mathematics, Comsats University, Islamabad, Islamabad, Pakistan

**Keywords:** Coronavirus model, Stochastic ordinary differential equations, Nonstandard computational method, Convergence analysis

## Abstract

The current effort is devoted to investigating and exploring the stochastic nonlinear mathematical pandemic model to describe the dynamics of the novel coronavirus. The model adopts the form of a nonlinear stochastic susceptible-infected-treated-recovered system, and we investigate the stochastic reproduction dynamics, both analytically and numerically. We applied different standard and nonstandard computational numerical methods for the solution of the stochastic system. The design of a nonstandard computation method for the stochastic system is innovative. Unfortunately, standard computation numerical methods are time-dependent and violate the structure properties of models, such as positivity, boundedness, and dynamical consistency of the stochastic system. To that end, convergence analysis of nonstandard computational methods and simulation with a comparison of standard computational methods are presented.

## Introduction

Humanity is enduring many diseases of variable lethality since its birth. Ebola, HIV, and Lassa fever are just a few of them. Ebola is a devastating disease that is transmitted to humans from carrier nonhuman primates, named fruit bats, and destroyed races of humankind. HIV is transferred from cross-species of chimpanzee to humans. In the earlier 19th century it was unknown. It spread rapidly to five continents of the world, killing 300,000 people, including children and women, because its signs and symptoms did not accompany any transmission. Lassa fever has its severity and history of destroying humankind. It is transmitted to humans via rats. Not only this fever, but many other diseases are also present that prove themselves devastating for the living being. Therefore, scientists tried very hard to build instruments to encounter the adverse effects of ailments and to produce possible treatment via vaccine or medicine. Among several other vicious diseases, COVID-19 has uprooted the humanity by killing many of people and is still consuming many lives to date. It was first discovered in the city Wuhan, Province Hubei in China [1, 2]. It is a pandemic that causes respiratory disorder and is transmitted through sneezing droplets of infected individuals. These droplets can fall on the objects around the effected and enter a healthy individual through contact. The number of cases is surging dramatically, raping developed and undeveloped countries together. It is frequently spreading so that it becomes impossible for the world’s aristocrats to overcome [3]. Currently, every continent is a sufferer, and among them, China, Iran, the UK, the USA, Spain, Italy are considered the most effected countries. Major symptoms of this disease include lethargy, dry cough, followed by fever [4]. Few patients may develop pains and aches, running nose with nasal congestion, diarrhea, and sore throat. Some individuals have developed these as mild symptoms, while others may show their severe forms. Those affected with mild symptoms recover through special treatment. Every individual out of six showed recovery. This virus shows severe symptoms in older individuals and in those who are already indulged with some sort of disease, like diabetes, cardiovascular disorders, cancer, chronic respiratory disorders, etc. The outbreak of COVID-19 raised many questions like how much time is required to formulate the vaccine or medicine to treat this disease effectively [5]. When would the world be free of all the deadly viruses? How much recession and life loss will occur, and will the world overcome this loss? No answer is available right now. Even though the recovery ratio is much larger compared to the death ratio, the number of fatalities is surging [6, 7]. Khan and Atangana [8] have suggested a fractional-order COVID-19 model with the Atangana–Baleanu–Caputo operator and have introduced this model to evaluate the infection in Wuhan. In the absence of efficient vaccines and treatment to mitigate the COVID-19 pandemic, the function of lock-down is studied [9]. In formulating the proposed mathematical model, the author used the new fractional operator’s method. In [10] Khan analyzes the dynamics of the current chaotic system, i.e., using Caputo–Fabrizio and Atangana–Baleanu derivatives. Some other articles on Atangana–Baleanu derivative applications can be found in [11, 12]. A coronavirus model has recently been mathematically considered in [13]. The authors used Pakistan’s actual data and discussed the potential control and infection removal from Pakistan. In [14], where potential removal of the virus was discussed, the data from Ghana and its study using a mathematical model were taken into consideration.

In this paper, we aim to suggest and present mathematical analysis revealing the spread of such a deathly disease, and develop some prediction with real-world data [15, 16]. Also, the proposed structure-preserving nonstandard computational method, named the stochastic nonstandard finite difference (SNSFD), is applied for the given pandemic model [17, 18]. This is the critical point of this paper. The flow of the paper is based on the following sections: In Sect. 2, the formulation of the deterministic susceptible–infected–treated–recovered model is given. In Sect. 3, we formulate the stochastic susceptible–infected–treated–recovered model and study its threshold dynamics. In Sect. 4, we present different numerical methods for the stochastic model and develop convergence analysis. Finally, in Sect. 5, the conclusion is presented.

## Formulation of deterministic SITR model

In this section, we consider the dynamics of a human population, which is divided into four components, or subpopulations. The population will be represented by $N:[0,\infty )\rightarrow \mathcal{R}$, as a function of the time $t\geq 0$. Furthermore, each of the components of the population will be denoted by a nonnegative differentiable function $S,I,T,R:[0,\infty )\rightarrow \mathcal{R}$. The components of the human population are described as follows: ${S}({t})$ denotes those who are not infected yet with the coronavirus, but have some serious diseases, $I ({t})$ denotes humans infected with the coronavirus, $T ({t})$ denotes those who are availed via the vaccination or precautionary measures or quarantine, and $R ({t})$ denotes those who have recovered from the coronavirus. The nonnegative constants of the model are presented as follows: *B* denotes the natural rate of new-borns or rate of individuals who have travelled from other countries, *β* is the rate of interaction between individuals with serious diseases and individuals infected with coronavirus, *δ* is the rate of individuals with serious diseases who have recovered directly from the coronavirus due to their immune system or quarantine or extensive use of precautions, *α* is the rate of individuals who may die due to coronavirus or naturally, *μ* is the rate of individuals infected with coronavirus who are treated, and *ρ* is the rate of treated individuals who are completely recovered due to quarantine or vaccination. The dynamics of the coronavirus model is given by the nonlinear system of coupled ordinary differential equations as follows:

1 $$\begin{aligned}& S ' (t)= B-\beta S (t) I (t) -\alpha S (t) -\delta S (t),\quad \forall t\geq 0, \end{aligned}$$

2 $$\begin{aligned}& I ' (t)= \beta S (t) I (t) -\alpha I (t) -\mu I (t),\quad \forall t \geq 0, \end{aligned}$$

3 $$\begin{aligned}& T ' (t)= \mu I (t) -\alpha T (t) +\delta S (t) -\rho T (t),\quad \forall t\geq 0, \end{aligned}$$

4 $$\begin{aligned}& R ' (t)= \rho T (t) -\alpha R (t),\quad \forall t\geq 0. \end{aligned}$$

Obviously, the identity $N(t)=S ( t ) +I ( t ) +T ( t ) +R(t)$ is satisfied at all time instances $t\geq 0$. Let $S_{0}$, $I_{0}$, $T_{0}$, and $R_{0}$ be nonnegative real numbers that represent the initial sizes of each component of the population, respectively. More clearly, the following conditions are satisfied:

5 $$ S_{0} =S ( 0 ),\qquad I_{0} =I ( 0 ),\qquad T_{0} =T ( 0 ),\qquad R_{0} =R ( 0 ). $$

### Basic properties

#### Lemma 1

*For any given nonnegative initial conditions*, *there exists a unique solution* *S*, *I*, *T*, *R*, *respectively*, *for all* ${t}\geq 0$. *Moreover*, *it satisfies the following inequality of boundedness*: $\lim_{{t}\longrightarrow \infty} \sup N (t)\leq \frac{B}{\alpha } $.

#### Proof

The total dynamics of the model (1)–(4) is obtained by adding the four equations as follows:

5a $$ \frac{d N}{dt} \leq B- \alpha N. $$

It follows that

5b $$ N(t)\leq N(0) e^{-\alpha t} + \frac{B}{\alpha } . $$

Thus a solution of the model (1)–(4) exists for given initial conditions and is eventually bounded on every finite time interval. □

#### Lemma 2

*The closed set* $\Gamma =\{S ( t ),E ( t ),T ( t ),R ( t ) \epsilon R_{+}^{4}: S ( t ) +I ( t ) +T ( t ) +R ( t ) \leq \frac{B}{\alpha } \}$*is positively invariant*.

#### Proof

Using Eqs. (5a) and (5b), it follows that as the time approaches infinity, ${t}\rightarrow \infty $, the population is bounded by a positive number so on the set Γ,

$$ N(t)\leq \frac{B}{\alpha }, $$

therefore, the set Γ is positively invariant. □

### Steady states of the model

It is easy to see that there are three steady states of Eqs. (1) to (4) as follows:

- $\mbox{Trivial equilibrium (TE)} = ( S, I,{T},{R} ) = (0,0,0,0)$;
- $\mbox{Virus-free state ({VFS})}= V_{1} = ( S^{0}, I^{0}, T^{0}, R^{0} ) = ( \frac{{B}}{\alpha +\delta },0,0,0)$;
- $\mbox{Virus existence state ({VES})}= V_{2} = ( S^{1}, I^{1}, T^{1}, R^{1} )$,

where

$$ S^{1} = \frac{\alpha +\mu }{\beta },\qquad I^{1} = \frac{{B}-(\alpha +\delta ) S^{1}}{\beta S^{1}},\qquad T^{1} = \frac{\mu I^{1} +\delta S^{1}}{\alpha +\rho },\qquad R^{1} = \frac{S T^{1}}{\alpha }. $$

Notice that the reproduction number $R_{O}$ is the spectral radius of $G_{1} G_{2}^{-1}$ [4], where

$$ G_{1} = \begin{bmatrix} \frac{{B}\beta }{\alpha +\delta } & 0 & 0\\ 0 & 0 & 0\\ 0 & 0 & 0 \end{bmatrix}\quad \text{and}\quad G_{2} = \begin{bmatrix} \alpha +\mu & 0 & 0\\ -\mu & \alpha +\delta & 0\\ 0 & -\rho & \alpha \end{bmatrix}. $$

More precisely, notice that

$$ R_{O} = \frac{B\beta }{ ( \alpha +\mu ) (\alpha +\delta )}. $$

## Stochastic SITR model

Let us consider the vector ${V}({t})= [ S(t),{I} (t),{T} (t),{R} (t) ]^{{T}}$, the possible changes in the given model are as presented in Table 1.

**Table 1:** Transition probabilities

${T}_{{i}}= \mbox{Transition}$	${P}_{{i}}= \mbox{Probabilities}$
${T}_{{2}} = [ -{1} \ {1} \ {0} \ {0} ]^{{T}}$	${P}_{2} = \beta {SI}\Delta {t}$
${T}_{{3}} = [ -{1} \ {0} \ {0} ]^{{T}}$	${P}_{3} = \alpha {S}\Delta {t}$
${T}_{{4}} = [ -{1} \ {0} \ {1}\ {0} ]^{{T}}$	${P}_{4} = \delta S \Delta {t}$
${T}_{{5}} = [ {0} \ -{1} \ {0}\ {0} ]^{{T}}$	${P}_{5} = \alpha {I} \Delta {t}$
${T}_{{6}} = [ {0} \ -{1} \ {1} \ {0} ]^{{T}}$	${P}_{6} = \mu I \Delta {t}$
${T}_{{7}} = [ {0}\ {0}\ -{1}\ {0} ]^{{T}}$	${P}_{7} = \alpha T \Delta {t}$
${T}_{{8}} = [ {0}\ {0}\ -{1} \ {1} ]^{{T}}$	${P}_{8} =\rho {T} \Delta {t}$
${T}_{{9}} = [ {0} \ {0} \ {0}\ -{1} ]^{{T}}$	${P}_{9} =\alpha {R} \Delta {t}$

Notice that the expectation and variance of the given model are as follows:

$$\begin{aligned}& {E}^{*} [ \Delta {V} ] = \sum_{{i}=1}^{9} {P}_{{i}} {T}_{{i}} = \begin{bmatrix} {B} -\beta SI-\alpha S-\delta S\\ \beta SI-\alpha I-\mu I\\ \mu {I} -\alpha T+\delta S-\rho T \\ \rho T-\alpha R \end{bmatrix} \Delta t, \\& \begin{aligned} \operatorname{Var} &= {E}^{*} \bigl[ \Delta {V} \Delta {V}^{{T}} \bigr] = \sum_{{i}=1}^{9} {P}_{{i}} [{T}_{{i}} ][{T}_{{i}} ]^{{T}} \\ &= \begin{bmatrix} P_{1} + P_{2} + P_{3} + P_{4} & - P_{2} & - P_{4} & 0\\ - P_{2} & P_{2} + P_{5} + P_{6} & - P_{6} & 0\\ - P_{4} & - P_{6} & P_{6} + P_{7} + P_{8} & -P_{8}\\ 0 & 0 & - P_{8} & P_{8} + P_{9} \end{bmatrix}. \end{aligned} \end{aligned}$$

Note that the stochastic drift is ${f} ( {V} ( {t} ),{t} ) = \frac{{E}^{*} [ \Delta {V} ]}{\Delta {t}} $, while stochastic diffusion is ${L} ( {V}({t}),{t} ) = \sqrt{\frac{{E}^{*} [\Delta {V} \Delta {V}^{{T}} ]}{\Delta {t}}} $.

So, the stochastic differential equation of the given model is as follows:

6 $$ {dV} ( {t} ) ={f} \bigl( {V}({t}),{t} \bigr) \,{dt}+{L} \bigl( {V}({t}),{t} \bigr) \,{dW}({t}), $$

with initial conditions ${V} ( 0 ) = {V}_{{o}} = [0.7,0.05, 0.2,0.05]^{{T}}$, $0\leq {t}\leq {T}$, and where the Brownian motion is denoted by ${W}({t})$.

### Euler–Maruyama method

This method can be applied to Eq. (6) as follows [19]:

7 $$ V_{m+1} = V_{m} +f ( V_{m},t ) \Delta t+L ( V_{m},t ) \Delta W_{m}, $$

where Δ*t* is the time step size and $\Delta {W}_{m} = {W}_{t_{m+1}} - {W}_{t_{m}}$ is a random variable having the standard normal distribution. It is normally distributed between stochastic drift and stochastic diffusion, i.e., $\Delta {W}_{m} \sim {N}(0, 1)$.

## Parametric noise in SITR model

In this section, we shall choose parameters from Eq. (1) to (4) and change them into random parameters with small noise as $\beta \,dt=\beta \,dt+ \sigma \,dW(t)$ as follows [20]:

8 $$\begin{aligned}& dS(t)= \bigl( {B} -\beta S (t) I (t) -\alpha S (t) -\delta S (t) \bigr) \,dt- \sigma I(t)S(t)\,dW(t),\quad \forall t\geq 0, \end{aligned}$$

9 $$\begin{aligned}& dI(t)=\bigl( \beta S (t) I (t) -\alpha I (t) -\mu I (t)\bigr)\,dt+ \sigma I(t)S(t)\,dW(t),\quad \forall t\geq 0, \end{aligned}$$

10 $$\begin{aligned}& dT (t) =\bigl( \mu {I} (t) -\alpha T (t) +\delta S (t) -\rho T (t) \bigr)\,dt, \quad \forall t\geq 0, \end{aligned}$$

11 $$\begin{aligned}& dR (t) =\bigl(\rho T (t) -\alpha R (t) \bigr)\,dt,\quad \forall t\geq 0. \end{aligned}$$

The Wiener process is denoted by $W_{k} ( t )$, while *σ* is the randomness of Eqs. (8) to (11). The system of Eqs. (8) to (11) is nonintegrable because of the Wiener process.

### Stochastic reproduction dynamics

Let us introduce ${R}_{{o}}^{{S}} = {R}_{{o}}^{{d}} - \frac{\sigma ^{2}}{2 ( \alpha +\mu )}$, where ${R}_{{o}}^{{S}}$ denotes the stochastic reproduction number.

#### Lemma 3

*If the initial data satisfy* $( {S} ( 0 ), {I} ( 0 ), {T} ( 0 ), {R} ( 0 ) ) \in {R}_{+}^{4}$, *then the system* (8)*–*(11) *has a unique solution*, *and the solution* $( {S} ( {t} ), I ( {t} ),{T} ( {t} ), {R} ( {t} ) )$*belongs to* Γ.

#### Definition

If $\lim_{{t}\rightarrow \infty } I ( {t} ) =0 $, then in the system (8)–(11), infected individuals will experience extinction.

#### Theorem 1

*If* ${R}_{{o}}^{{S}} < 1$*and* $\sigma ^{2} < \frac{B}{(\alpha +\delta )}$, *then the number of infected individuals in the system* (8)*–*(11) *exponentially tends to zero*.

#### Proof

Assume that initial data satisfy $( {S} ( 0 ),{I} ( 0 ),{T} ( 0 ), {R} ( 0 ) ) \in {R}_{+}^{4}$ and $( {S} ( {t} ), I ( {t} ),{T} ( {t} ), {R} ( {t} ) )$ is a solution of system (8)–(11), with constant randomness *σ* and constant drift *β*, satisfying the stochastic differential equation $dI=( \beta SI-\alpha I-\mu I )\,dt+ \sigma IS\,dW$ for a Brownian motion *W*. Applying Itô’s lemma with ${f} (I )= \ln ({I})$ gives

$$\begin{aligned}& {dln} ( I ) = {f}' ( {I} ) \,{dI}+ \frac{1}{2} {f}^{\prime \prime } ( {I} ) {I}^{2} \sigma ^{2} \,{dt}, \\& {d} \ln (I )= \frac{1}{{I}} \,{dI}+ \frac{1}{2} \biggl(- \frac{1}{{I}^{2}} \biggr) {I}^{2} \sigma ^{2} \,{dt}, \\& {d} \ln (I )=\biggl( \beta S-\alpha -\mu - \frac{1}{2} \sigma ^{2} \biggr)\,{dt}+\sigma {S\,dW}. \end{aligned}$$

More precisely, by integrating from $[0,t]$, $\forall t\geq 0$,

$$ \ln ( I )= \ln {I} ( 0 ) + \int _{0}^{{t}} \biggl( \beta S-\alpha -\mu - \frac{1}{2} \sigma ^{2} \biggr)\,{dt}+ \int _{0}^{{t}} \sigma {S\,dW}. $$

Notice that a local continuous martingale is defined as ${M} ( {t} ) = \int _{0}^{{t}} \sigma {S\,dW}$ with ${M} ( 0 ) =0$.

If $\sigma ^{2} > \frac{B\beta }{ ( \alpha +\mu ) (\alpha +\delta )}$,

$$\begin{aligned}& \ln ( I ) >\biggl( \frac{\beta B}{(\alpha +\delta )} -( \alpha +\mu )- \frac{1}{2} \frac{B\beta }{ ( \alpha +\mu ) (\alpha +\delta )} \biggr){t}+{M} ( {t} ) + \ln I ( 0 ), \\& \frac{\ln (I )}{{t}} > \biggl( \frac{B\beta (2 ( \alpha +\mu ) -1 )}{2 ( \alpha +\mu ) (\alpha +\delta )} -( \alpha +\mu ) \biggr) + \frac{{M} ( {t} )}{{t}} + \frac{\ln I ( 0 )}{{t}}, \\& \lim_{{t}\rightarrow \infty } \frac{\ln (I )}{{t}} > \biggl( \frac{B\beta (2 ( \alpha +\mu ) -1 )}{2 ( \alpha +\mu ) (\alpha +\delta )} -( \alpha +\mu ) \biggr) >0,\quad \text{with } \lim_{{t}\rightarrow \infty } \frac{{M} ( {t} )}{{t}} =0, \end{aligned}$$

If $\sigma ^{2} < \frac{B\beta }{ ( \alpha +\mu ) (\alpha +\delta )}$, then

$$\begin{aligned}& \ln \bigl( I ( {t} ) \bigr) < \biggl( \frac{\beta B}{(\alpha +\delta )} -( \alpha +\mu )- \frac{1}{2} \sigma ^{2} \biggr) {t}+{M} ( {t} ) + \ln I ( 0 ), \\& \frac{\ln (I )}{{t}} < ( \alpha +\mu ) \biggl( \frac{\beta B}{ ( \alpha +\delta ) ( \alpha +\mu )} - \frac{\sigma ^{2}}{2 ( \alpha +\mu )} -1 \biggr) + \frac{{M} ( {t} )}{{t}} + \frac{\ln I ( 0 )}{{t}}, \\& \lim_{{t}\rightarrow \infty } \sup \frac{\ln (I )}{{t}} < ( \alpha +\mu ) \bigl( {R}_{0}^{{S}} -1 \bigr), \end{aligned}$$

so that, when

$${R}_{0}^{{S}} < 1, $$

we get

$$\begin{aligned}& \lim_{{t}\rightarrow \infty } \sup \frac{\ln (I )}{{t}} \leq 0, \\& \lim_{{t}\rightarrow \infty } I ( {t} ) =0, \end{aligned}$$

as desired. Moreover,

$$ {R}_{{o}}^{{S}} = {R}_{{o}}^{{d}} - \frac{\sigma ^{2}}{2 ( \alpha +\mu )} < 1. $$

 □

## Numerical methodology

For each $N\epsilon \mathbb{N}$, define the set $I_{N} = \{ 0,1,2,\dots ,N \} $. In this section, we will provide and analyze a discretization of the system (8)–(11). To that end, we consider the temporal period of length $T>0$. Fix a uniform partition of the temporal interval $[ 0,T ]$ consisting of *N* subintervals, and let $k= \frac{T}{N}$. Defining $t_{m} =mk$, for each $m\epsilon I_{N}$, we will employ the notation $S^{m}$, $I^{m}$, $T^{m}$, and $R^{m}$ to represent the numerical approximation to the values of the functions *S*, *I*, *T*, and *R*, respectively, at time $t_{m}$.

Also, we will apply different explicit and implicit methods to the given system. As expected, we will employ the discrete initial data $( S_{0}, I_{0}, T_{0}, R_{0} )$, where $S_{0} =S ( 0 )$, $I_{0} =I ( 0 )$, $T_{0} =T ( 0 )$, $R_{0} =R ( 0 )$.

### Stochastic Euler method

This method can be applied to the system of Eqs. (8) to (11) as follows [21]:

12 $$\begin{aligned}& S^{m+1} = S^{m} +k\bigl( {B} -\beta S^{m} I^{m} -\alpha S^{m} -\delta S^{m} - S^{m} I^{m} \sigma \Delta W_{m} \bigr), \end{aligned}$$

13 $$\begin{aligned}& I^{m+1} = I^{m} +k\bigl(\beta S^{m} I^{m} -\alpha I^{m} -\mu I^{m} + S^{m} I^{m} \sigma \Delta W_{m} \bigr), \end{aligned}$$

14 $$\begin{aligned}& T^{m+1} = T^{m} +k\bigl( \mu {I}^{m} -\alpha T^{m} +\delta S^{m} -\rho T^{m} \bigr), \end{aligned}$$

15 $$\begin{aligned}& R^{m+1} = R^{m} +k\bigl(\rho T^{m} -\alpha R^{m} \bigr), \end{aligned}$$

where *k* is time step size and $\Delta {W}_{m} = {W}_{t_{m+1}} - {W}_{t_{m}}$.

### Stochastic Runge–Kutta method

This method can be applied to the system of Eqs. (8) to (11) as follows:

Stage 1

$$\begin{aligned}& A_{1} = k\bigl( {B} -\beta S^{m} I^{m} -\alpha S^{m} -\delta S^{m} - S^{m} I^{m} \sigma \Delta W_{m} \bigr), \\& B_{1} = k\bigl(\beta S^{m} I^{m} -\alpha I^{m} -\mu I^{m} + S^{m} I^{m} \sigma \Delta W_{m} \bigr), \\& C_{1} =k\bigl( \mu {I}^{m} -\alpha T^{m} +\delta S^{m} -\rho T^{m} \bigr), \\& D_{1} =k\bigl(\rho T^{m} -\alpha R^{m} \bigr). \end{aligned}$$

Stage 2

$$\begin{aligned}& \begin{aligned} A_{2} &= k \biggl[ {B} -\beta \biggl( S^{m} + \frac{A_{1}}{2} \biggr) \biggl( I^{m} + \frac{B_{1}}{2} \biggr) - ( \alpha +\delta ) \biggl( S^{m} + \frac{A_{1}}{2} \biggr) \\ &\quad {}- \biggl( S^{m} + \frac{A_{1}}{2} \biggr) \biggl( I^{m} + \frac{B_{1}}{2} \biggr) \sigma \Delta W_{m} \biggr], \end{aligned} \\& \begin{aligned} B_{2} &= k \biggl[ \beta \biggl( S^{m} + \frac{A_{1}}{2} \biggr) \biggl( I^{m} + \frac{B_{1}}{2} \biggr) - ( \alpha +\mu ) \biggl( I^{m} + \frac{B_{1}}{2} \biggr) \\ &\quad {}+ \biggl( S^{m} + \frac{A_{1}}{2} \biggr) \biggl( I^{m} + \frac{B_{1}}{2} \biggr) \sigma \Delta W_{m} \biggr], \end{aligned} \\& C_{2} =k \biggl[ \mu \biggl( I^{m} + \frac{B_{1}}{2} \biggr) -\alpha \biggl( T^{m} + \frac{C_{1}}{2} \biggr) +\delta \biggl( S^{m} + \frac{A_{1}}{2} \biggr) -\rho \biggl( T^{m} + \frac{C_{1}}{2} \biggr) \biggr], \\& D_{2} =k \biggl[ \rho \biggl( T^{m} + \frac{C_{1}}{2} \biggr) -\alpha \biggl( R^{m} + \frac{D_{1}}{2} \biggr) \biggr]. \end{aligned}$$

Stage 3

$$\begin{aligned}& \begin{aligned} A_{3} &= k \biggl[ {B} -\beta \biggl( S^{m} + \frac{A_{2}}{2} \biggr) \biggl( I^{m} + \frac{B_{2}}{2} \biggr) - ( \alpha +\delta ) \biggl( S^{m} + \frac{A_{2}}{2} \biggr)\\ &\quad {} - \biggl( S^{m} + \frac{A_{2}}{2} \biggr) \biggl( I^{m} + \frac{B_{2}}{2} \biggr) \sigma \Delta W_{m} \biggr], \end{aligned} \\& \begin{aligned} B_{3} &= k \biggl[ \beta \biggl( S^{m} + \frac{A_{2}}{2} \biggr) \biggl( I^{m} + \frac{B_{2}}{2} \biggr) - ( \alpha +\mu ) \biggl( I^{m} + \frac{B_{2}}{2} \biggr) \\ &\quad {}+ \biggl( S^{m} + \frac{A_{2}}{2} \biggr) \biggl( I^{m} + \frac{B_{2}}{2} \biggr) \sigma \Delta W_{m} \biggr], \end{aligned} \\& C_{3} =k \biggl[ \mu \biggl( I^{m} + \frac{B_{2}}{2} \biggr) -\alpha \biggl( T^{m} + \frac{C_{2}}{2} \biggr) +\delta \biggl( S^{m} + \frac{A_{2}}{2} \biggr) -\rho \biggl( T^{m} + \frac{C_{2}}{2} \biggr) \biggr], \\& D_{3} =k \biggl[ \rho \biggl( T^{m} + \frac{C_{2}}{2} \biggr) -\alpha \biggl( R^{m} + \frac{D_{2}}{2} \biggr) \biggr]. \end{aligned}$$

Stage 4

$$\begin{aligned}& A_{4} = k \bigl[ {B} -\beta \bigl( S^{m} + A_{3} \bigr) \bigl( I^{m} + B_{3} \bigr) - ( \alpha +\delta ) \bigl( S^{m} + A_{3} \bigr) - \bigl( S^{m} + A_{3} \bigr) \bigl( I^{m} + B_{3} \bigr) \sigma \Delta W_{m} \bigr], \\& B_{4} = k \bigl[ \beta \bigl( S^{m} + A_{3} \bigr) \bigl( I^{m} + B_{3} \bigr) - ( \alpha +\mu ) \bigl( I^{m} + B_{3} \bigr) + \bigl( S^{m} + A_{3} \bigr) \bigl( I^{m} + B_{3} \bigr) \sigma \Delta W_{m} \bigr], \\& C_{4} =k \bigl[ \mu \bigl( I^{m} + B_{3} \bigr) -\alpha \bigl( T^{m} + C_{3} \bigr) +\delta \bigl( S^{m} + A_{3} \bigr) -\rho \bigl( T^{m} + C_{3} \bigr) \bigr], \\& D_{4} =k \bigl[ \rho \bigl( T^{m} + C_{3} \bigr) -\alpha \bigl( R^{m} + D_{3} \bigr) \bigr]. \end{aligned}$$

Final stage

16 $$ \left . \textstyle\begin{array}{l} {S}^{{n}+1} = {S}^{{n}} + \frac{1}{6} [ {A}_{1} +2 {A}_{2} +2 {A}_{3} + {A}_{4} ],\\ {I}^{{n}+1} = {I}^{{n}} + \frac{1}{6} [ {B}_{1} +2 {B}_{2} +2 {B}_{3} + {B}_{4} ],\\ {T}^{{n}+1} = {T}^{{n}} + \frac{1}{6} [ {C}_{1} +2 {C}_{2} +2 {C}_{3} + {C}_{4} ], \\ {R}^{{n}+1} = {R}^{{n}} + \frac{1}{6} [ {D}_{1} +2 {D}_{2} +2 {D}_{3} + {D}_{4} ], \end{array}\displaystyle \right \} $$

where *k* is the time step size and $\Delta {W}_{m} = {W}_{t_{m+1}} - {W}_{t_{m}}$.

### Nonstandard computational method

This method can be applied to the system of Eqs. (8) to (11) as follows:

17 $$\begin{aligned}& S^{m+1} = \frac{S^{m} +k {B}}{1+k\beta I^{m} +k ( \alpha +\delta ) +k I^{m} \sigma \Delta W_{m}}, \end{aligned}$$

18 $$\begin{aligned}& I^{m+1} = \frac{I^{m} + k\beta S^{m} I^{m} +k S^{m} I^{m} \sigma \Delta W_{m}}{1+k ( \alpha +\mu )}, \end{aligned}$$

19 $$\begin{aligned}& T^{m+1} = \frac{T^{m} + k \mu {I}^{m} +k\delta S^{m}}{1+k ( \alpha +\rho )}, \end{aligned}$$

20 $$\begin{aligned}& R^{m+1} = \frac{R^{m} + k \rho {T}^{m}}{1+k\alpha }, \end{aligned}$$

where *k* is the time step size and $\Delta {W}_{m} = {W}_{t_{m+1}} - {W}_{t_{m}}$.

#### Convergence analysis

In this section, we shall present the following theorems for positivity, boundedness, consistency, and stability.

##### Theorem 2

*For any given initial value* $( {S}^{{m}} (0), {I}^{{m}} (0), {T}^{{m}} (0), {R}^{{m}} (0)) \in {R}_{+}^{4}$, *the system of Eqs*. (17) *to* (20) *has a unique positive solution* $( {S}^{{m}}, {I}^{{m}}, {T}^{{m}}, {R}^{{m}} ) \in {R}_{+}^{4}$*for any* $m \geq 0$.

##### Proof

All the parameters and initial conditions must be nonnegative due to biological reasoning. The proof is straightforward. □

##### Theorem 3

*The region* $\Gamma = \{ ( {S}^{{m}}, {I}^{{m}}, {T}^{{m}}, {R}^{{m}} ) \in {R}_{+}^{4}: {S}^{{m}} \geq 0, {I}^{{m}} \geq 0, {T}^{{m}} \geq 0, {R}^{{m}} \geq 0, {S}^{{m}} + {I}^{{m}} + {T}^{{m}} + {R}^{{m}} \leq \frac{{B}}{\alpha } \} $*for all* ${m}\geq 0$*is a positively invariant feasible region for Eqs*. (17) *to* (20).

##### Proof

We rewrite the system (17) to (20) as follows:

$$\begin{aligned}& \frac{{S}^{{m}+1} - {S}^{{m}}}{{k}} = \bigl( {B} -\beta S^{m} I^{m} -\alpha S^{m} -\delta S^{m} - S^{m} I^{m} \sigma \Delta W_{m} \bigr), \\& \frac{{I}^{{m}+1} - {I}^{{m}}}{{k}} = \bigl( \beta S^{m} I^{m} -\alpha I^{m} -\mu I^{m} + S^{m} I^{m} \sigma \Delta W_{m} \bigr), \\& \frac{{T}^{{m}+1} - {T}^{{m}}}{{k}} = \bigl( \mu {I}^{m} -\alpha T^{m} + \delta S^{m} -\rho T^{m} \bigr), \\& \frac{{R}^{{m}+1} - {R}^{{m}}}{{k}} = \bigl( \rho T^{m} -\alpha R^{m} \bigr), \\& \frac{({S}^{{m}+1} + {I}^{{m}+1} + {T}^{{m}+1} + {R}^{{m}+1} )-( {S}^{{m}} + {I}^{{m}} + {T}^{{m}} + {R}^{{m}} )}{{k}} ={B}- \alpha \bigl( {S}^{{m}} + {I}^{{m}} + {T}^{{m}} + {R}^{{m}} \bigr), \\& \bigl({S}^{{m}+1} + {I}^{{m}+1} + {T}^{{m}+1} + {R}^{{m}+1} \bigr)= \bigl( {S}^{{m}} + {I}^{{m}} + {T}^{{m}} + {R}^{{m}} \bigr) +{kB}-{k} \alpha \bigl( {S}^{{m}} + {I}^{{m}} + {T}^{{m}} + {R}^{{m}} \bigr), \\& \bigl({S}^{{m}+1} + {I}^{{m}+1} + {T}^{{m}+1} + {R}^{{m}+1} \bigr)\leq \frac{{B}}{\alpha } +{kB}-{k} \alpha \biggl( \frac{{B}}{\alpha } \biggr), \\& \bigl({S}^{{m}+1} + {I}^{{m}+1} + {T}^{{m}+1} + {R}^{{m}+1} \bigr)\leq \frac{{B}}{\alpha }. \end{aligned}$$

 □

##### Theorem 4

*For any* $m\geq 0$, *the system of discrete dynamical Eqs*. (17) *to* (20) *has the same steady states as that of the continuous dynamical Eqs*. (8) *to* (11).

##### Proof

Solving the Eqs. (17) to (20), we get three states as follows:

- $\mbox{Trivial equilibrium (TE)} = ( S^{m}, I^{m}, T^{m}, R^{m} ) = (0,0,0,0)$,
- $\mbox{Virus-free state ({VFS})}= ( S^{m}, I^{m}, T^{m}, R^{m} ) = ( \frac{{B}}{\alpha +\delta },0,0,0 )$,
- $\mbox{Virus Existence state ({VES})}= ( S^{m}, I^{m}, T^{m}, R^{m} )$,

where

$$ S^{m} = \frac{\alpha +\mu }{\beta },\qquad I^{m} = \frac{{B}-(\alpha +\delta ) S^{m}}{\beta S^{m}},\qquad T^{m} = \frac{\mu I^{m} +\delta S^{m}}{\alpha +\rho },\qquad R^{m} = \frac{S T^{m}}{\alpha }. $$

 □

##### Theorem 5

*For any* $m\geq 0$, *the proposed computational method is stable if the eigenvalues of Eqs*. (17) *to* (20) *lie inside the unit circle*.

##### Proof

Consider the right-hand sides of Eqs. (17)–(20) as functions *F*, *G*, *H*, and *L* as follows:

$$\begin{aligned}& F= \frac{S+k {B}}{1+k\beta I+k ( \alpha +\delta ) +kI\sigma \Delta w_{m}},\qquad G= \frac{I+ k\beta SI+kSI \sigma \Delta w_{m}}{1+k ( \alpha +\mu )},\\& H= \frac{T+ k \mu {I} +k\delta S}{1+k ( \alpha +\rho )}, \qquad L= \frac{R+ k \rho {T}}{1+k\alpha }. \end{aligned}$$

The Jacobian matrix is defined as

$$ J= \begin{bmatrix} \frac{\partial F}{\partial S} & \frac{\partial F}{\partial I} & \frac{\partial F}{\partial T} & \frac{\partial F}{\partial R}\\ \frac{\partial G}{\partial S} & \frac{\partial G}{\partial I} & \frac{\partial G}{\partial T} & \frac{\partial G}{\partial R}\\ \frac{\partial H}{\partial S} & \frac{\partial H}{\partial I} & \frac{\partial H}{\partial T} & \frac{\partial H}{\partial R}\\ \frac{\partial L}{\partial S} & \frac{\partial L}{\partial I} & \frac{\partial L}{\partial T} & \frac{\partial L}{\partial R} \end{bmatrix} $$

where

$$\begin{aligned}& \frac{\partial F}{\partial S} = \frac{1}{1+k\beta I+k ( \alpha +\delta ) +kI\sigma \Delta W_{m}},\qquad \frac{\partial F}{\partial I} = \frac{-(S+k {B})[{k}\beta + k\sigma \Delta W_{m} ]}{[1+k\beta I+k ( \alpha +\delta ) +kI\sigma \Delta W_{m} ]^{2}},\\& \frac{\partial F}{\partial T} =0,\qquad \frac{\partial F}{\partial R} =0, \\& \frac{\partial G}{\partial S} = \frac{k\beta I+kI \sigma \Delta W_{m}}{1+k ( \alpha +\mu )},\qquad \frac{\partial G}{\partial I} = \frac{k\beta S+kS \sigma \Delta W_{m}}{1+k ( \alpha +\mu )},\qquad \frac{\partial G}{\partial T} =0,\qquad \frac{\partial G}{\partial R} =0, \\& \frac{\partial H}{\partial S} = \frac{k\delta }{1+k ( \alpha +\rho )},\qquad \frac{\partial H}{\partial I} = \frac{k\mu }{1+k ( \alpha +\rho )},\qquad \frac{\partial H}{\partial T} = \frac{1}{1+k ( \alpha +\rho )},\qquad \frac{\partial H}{\partial R} =0, \\& \frac{\partial L}{\partial S} =0,\qquad \frac{\partial L}{\partial I} =0, \qquad \frac{\partial L}{\partial T} = \frac{k\rho }{1+k\alpha },\qquad \frac{\partial L}{\partial R} = \frac{1}{1+k\alpha }. \end{aligned}$$

Now we want to linearize the model about the steady-state of the model for virus-free state $V_{1} =( \frac{{B}}{\alpha +\delta }, 0, 0, 0)$ and $R_{o} <1$.

The given Jacobian there is

$$ J( V_{1} )= \begin{bmatrix} \frac{1}{1+k ( \alpha +\delta )} & \frac{-( \frac{{B}}{\alpha +\delta } +k {B})[{k}\beta + k\sigma \Delta W_{m} ]}{[1+k ( \alpha +\delta ) ]^{2}} & 0 & 0 \\ 0 & \frac{k\beta ( \frac{{B}}{\alpha +\delta } )+k( \frac{{B}}{\alpha +\delta } ) \sigma \Delta w_{n}}{1+k ( \alpha +\mu )} & 0 & 0\\ \frac{k\delta }{1+k ( \alpha +\rho )} & \frac{k\mu }{1+k ( \alpha +\rho )} & \frac{1}{1+k ( \alpha +\rho )} & 0\\ 0 & 0 & \frac{k\rho }{1+k\alpha } & \frac{1}{1+k\alpha } \end{bmatrix}. $$

The eigenvalues of this Jacobian matrix are

$$\begin{aligned}& \lambda _{1} = \biggl\vert \frac{1}{1+k\alpha } \biggr\vert < 1, \qquad \lambda _{2} = \biggl\vert \frac{1}{1+k(\alpha +\rho )} \biggr\vert < 1,\\& \lambda _{3} = \biggl\vert \frac{1}{1+k(\alpha +\delta )} \biggr\vert < 1,\qquad \lambda _{4} = \frac{k\beta ( \frac{{B}}{\alpha +\delta } )+k( \frac{{B}}{\alpha +\delta } ) \sigma \Delta w_{n}}{1+k ( \alpha +\mu )} < 1,\quad \text{if } {R}_{{o}}^{{S}} < 1. \end{aligned}$$

**Figure 1:**
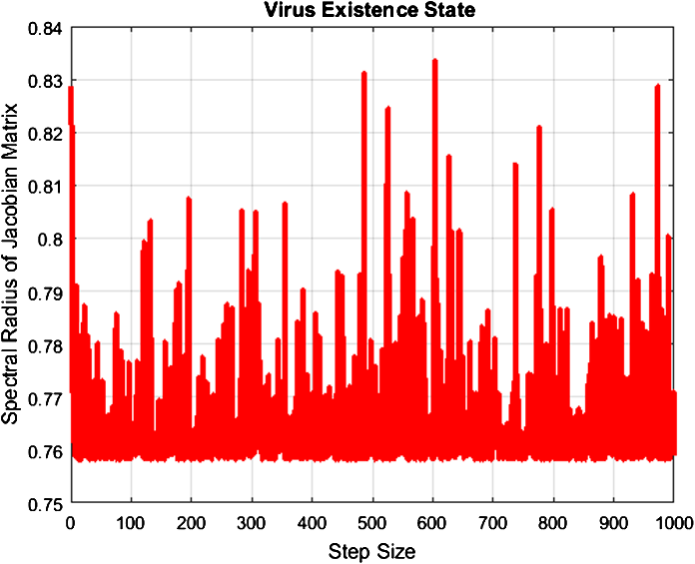
Graph of the spectral radius of the Jacobian matrix associated with the computational method (17)–(20) at the steady-state $V_{2}$. The estimated parameter values of Table 1 were used, and the spectral radius was obtained for various temporal time step sizes *k* in $[0,1000]$

So, the given model is stable around $V_{2}$ as shown in Fig. 1. This shows that all the eigenvalues of the Jacobian matrix lie inside the unit circle. So, the given model is stable around ${V}_{1}$. We plot the largest eigenvalues of the Jacobian matrix associated with $V_{2}$. Notice that the spectral radius is always less than one, as desired.

□

### Computational results

In this section, we present a comparison analysis of existing numerical methods with the nonstandard computational method by using different values of the parameters as presented in [22], ${B} =0.3$, $\beta =0.3$, $\alpha =0.3$, $\delta =0.001$, $\rho =0.03$, $\sigma =0.2$, for $R_{0}^{S} <1$. For $R_{0}^{S} >1$, ${B} =0.3$, $\beta =0.5$, $\alpha =0.3$, $\delta =0.001$, $\rho =0.03$, $\sigma =0.2$. Moreover, we used $S_{0} =0.7$, $I_{0} =0.05$, $T_{0} =0.2$, $R_{0} =0.05$.

#### Example 1

(Simulation for the free of coronavirus state)

**Figure 2:**
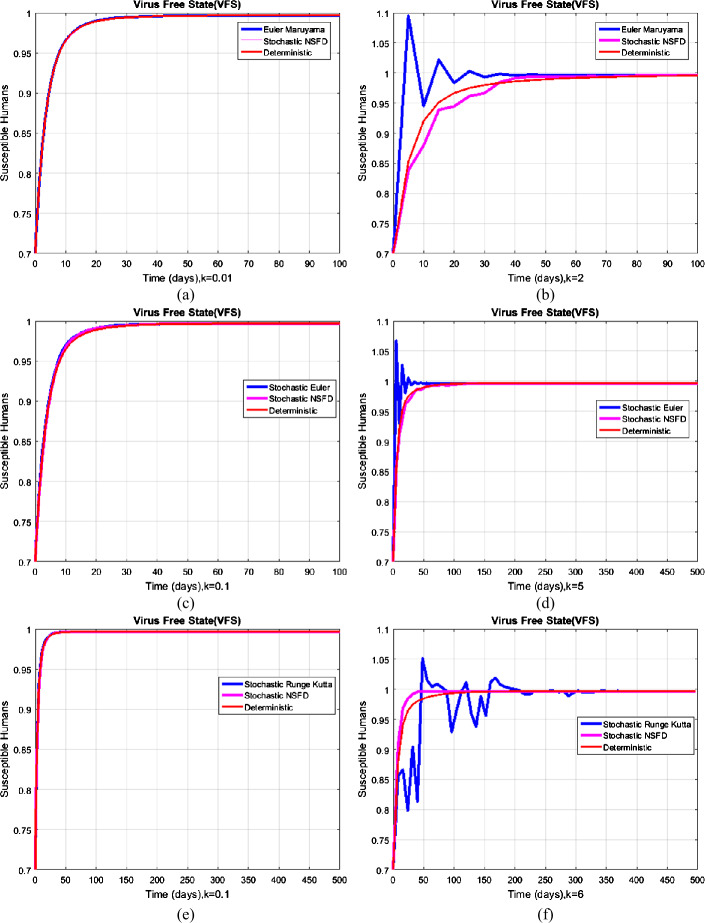
Comparison graphs of computational methods at the free of coronavirus state

For given real data, the reproductive value is $R_{0}^{S} = 0.6975 < 1$. So, the system converges to $V_{1} = ( \frac{{B}}{\alpha +\delta },0,0,0)$. However, in Fig. 2, we just take a susceptible component; the numerical solution of the system using the nonstandard and standard computational methods is presented. In Fig. 2, (a), (c), and (e), the results converge to $V_{1}$ for certain small temporal step size. On the other hand, in Fig. 2, (b), (d), and (f), the results violated the structural properties of the system. The comparison graph exhibits the fact that the proposed nonstandard computational method is capable of preserving the structural properties of the solutions in the sense of biological reasoning, as desired.

#### Example 2

(Simulation for the existence of coronavirus state)

**Figure 3:**
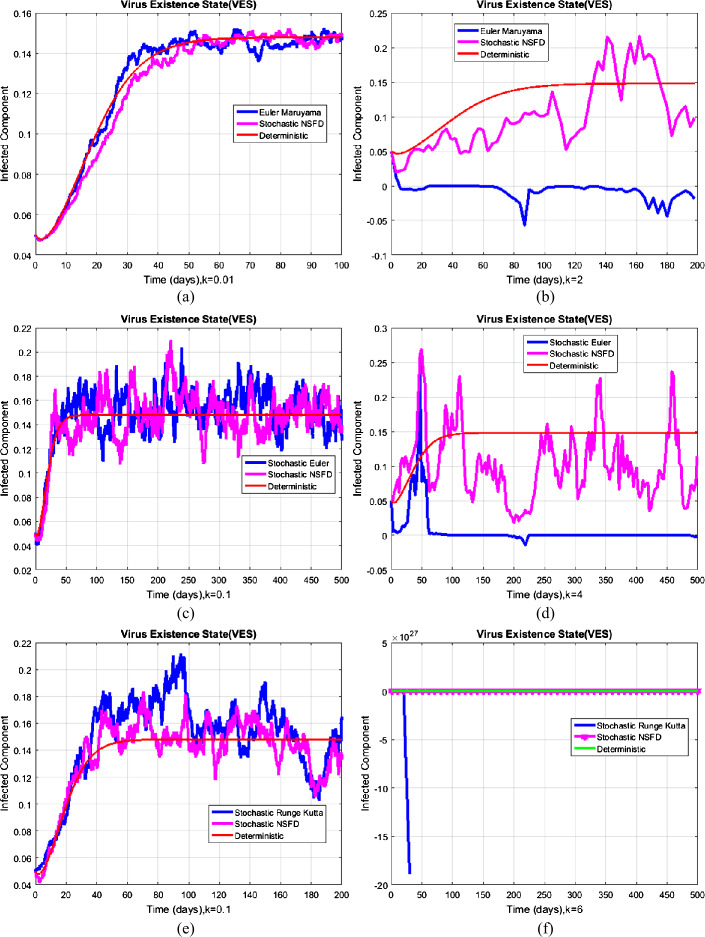
Comparison graph of computational methods at the existence of coronavirus state

**Figure 4:**
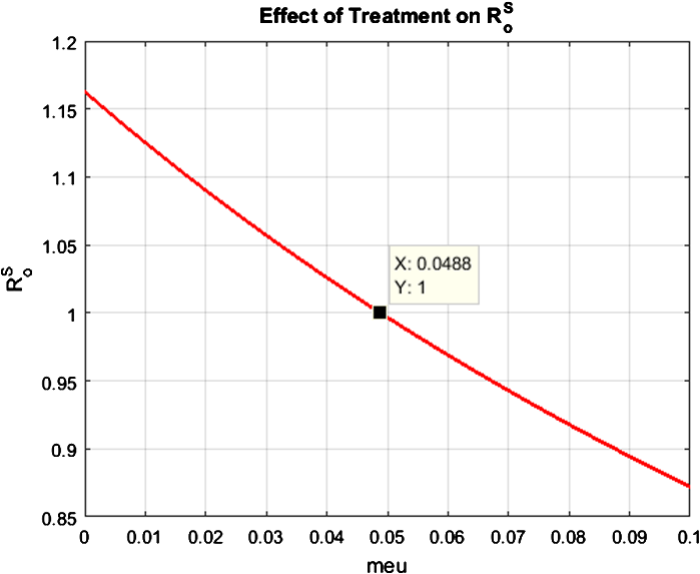
Graph of the effect of treatment on reproduction number $R_{0}^{S}$

The reproductive value is $R_{0}^{S} = 1.1958 > 1$. So, the system converges to $V_{2} =(0.8000, 0.1480, 0.0473,0.0047)$. Nevertheless, in Fig. 4, we just take the infected component; the numerical solution of the system using the nonstandard and standard computational methods is presented. In Fig. 3, (a), (c), and (e), the results converge to $V_{2}$ for a certain small temporal step size. On the other hand, in Fig. 3, (b), (d), and (f), the results violated the structural properties of the system. Figure 3 exhibits the fact that the nonstandard computational method has kept the structural properties of the model such as positivity, boundedness, and dynamical consistency, as desired.

#### Example 3

(Simulation for reproduction number with the effect of treatment)

Let $\mu =0.0488$. Notice that the reproduction value decreases, moving the dynamics of the model from virus existence state to virus-free state. So, the virus-free state of the system is stable. However, Fig. 4 exhibits the fact that the increases in treatment strategy such as quarantine or vaccination can overcome the pandemic of coronavirus, as desired.

#### Example 4

(Simulation for a component of the infected humans)

**Figure 5:**
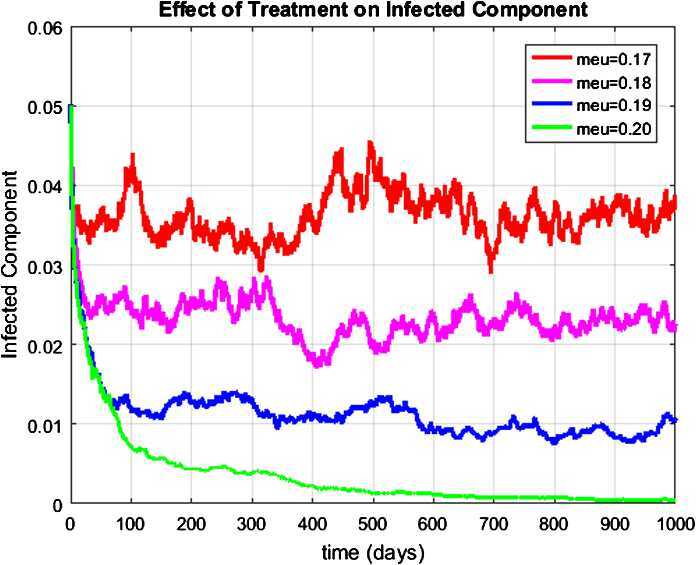
Graph of the effect of quarantine on infected humans at the existence of a virus state

Let us now for different values of *μ* (treatment rate) notice that the number of infected humans converges to zero. Eventually, the reported rate of infected humans has been controlled in certain scenarios. Consequently, Fig. 5 shows the fact that the treatment strategy has a vital role in the control of the pandemic of the coronavirus around the world.

## Conclusion

This study focused on the stochastic susceptible–infected–treated–recovered model, which elaborates a simplified way to describe the dynamics of coronavirus in the human population. Also, we have applied different explicit and implicit computational methods to study the dynamics of the stochastic system. The proposed nonstandard computational method is unconditionally convergent as compared to other explicit numerical methods. This method preserves the structural properties of stochastic models, such as consistency, stability, positivity, and boundedness [12]. Moreover, this study provides a valuable proof that the confinement rules are vital to control the situation in a reasonable time. If the contact rate is dropped between people then stabilization of classes can be achieved significantly earlier. Therefore, there is no chance for governments to get rid of social distancing rules among individuals.
